# Verses by Dr. Evans

**Published:** 1896-05

**Authors:** William J. Evans


### Editorial, Etc.

After the commencement of the Baltimore College of Dental Surgery, Friday night, March 20, 1896, the following verses were repeated to one of the graduates by the author, Dr. William J. Evans of New York. The concluding lines so impressed those who heard them that Dr. Evans was requested to commit the whole to paper, and they are printed as written at the moment, in pencil. We found in Dr. Evans a congenial friend. R. G.

The earth moves not in circles true,
It latitude evades;
And colors have a different hue,
In different lights and shades.

There is no measure for a flower,
No morrow like to day;
No birth, no school, nor any dower,
Can make us good, alway.

But each within his castle wall
Must find his holy grail;
There, he must learn how not to fall,
When what was strong is frail.

March 20th, 1896. WILLIAM J. EVANS.

